# Development and Characterization of Thermosensitive and Bioadhesive Ophthalmic Formulations Containing Flurbiprofen Solid Dispersions

**DOI:** 10.3390/gels10040267

**Published:** 2024-04-15

**Authors:** Pınar Adısanoğlu, Işık Özgüney

**Affiliations:** Department of Pharmaceutical Technology, Faculty of Pharmacy, University of Ege, 35100 Bornova, İzmir, Türkiye; pinar_adisan@hotmail.com

**Keywords:** in situ gelling, ophthalmic drug delivery, solid dispersion, poloxamer, flurbiprofen

## Abstract

In this study, we aimed to develop thermosensitive and bioadhesive in situ gelling systems containing solid dispersions of flurbiprofen (FB-SDs) using poloxamer 407 (P407) and 188 (P188) for ophthalmic delivery. FB-SDs were prepared with the melt method using P407, characterized by solubility, stability, SEM, DSC, TGA, and XRD analyses. Various formulations of poloxamer mixtures and FB-SDs were prepared using the cold method and P407/P188 (15/26.5%), which gels between 32 and 35 °C, was selected to develop an ophthalmic in situ gelling system. Bioadhesive polymers Carbopol 934P (CP) or carboxymethyl cellulose (CMC) were added in three concentrations (0.2, 0.4, and 0.6% (*w*/*w*)). Gelation temperature and time, mechanical properties, flow properties, and viscosity values were determined. The in vitro release rate, release kinetics, and the release mechanism of flurbiprofen (FB) from the ophthalmic formulations were analyzed. The results showed that FB-SDs’ solubility in water increased 332-fold compared with FB. The oscillation study results indicated that increasing bioadhesive polymer concentrations decreased gelation temperature and time, and formulations containing CP gel at lower temperatures and in a shorter time. All formulations except F3 and F4 showed Newtonion flow under non-physiological conditions, while all formulations exhibited non-Newtonion pseudoplastic flow under physiological conditions. Viscosity values increased with an increase in bioadhesive polymer concertation at physiological conditions. Texture profile analysis (TPA) showed that CP-containing formulations had higher hardness, compressibility, and adhesiveness, and the gel structure of formulation F4, containing 0.6% CP, exhibited the greatest hardness, compressibility, and adhesiveness. In vitro drug release studies indicated that CP and CMC had no effect below 0.6% concentration. Kinetic evaluation favored first-order and Hixson–Crowell kinetic models. Release mechanism analysis showed that the *n* values of the formulations were greater than 1 except for formulation F5, suggesting that FB might be released from the ophthalmic formulations by super case II type diffusion. When all the results of this study are evaluated, the in situ gelling formulations prepared with FB-SDs that contained P407/P188 (15/26.5%) and 0.2% CP or 0.2% CMC or 0.4 CMC% (F2, F5, and F6, respectively) could be promising formulations to prolong precorneal residence time and improve ocular bioavailability of FB.

## 1. Introduction

In situ gelling systems are liquid aqueous polymer solutions, and they can be conveniently administered into the conjunctival sac, where they undergo a transformation, transitioning from a liquid state to a gel [1,2]. The gelation can occur with several mechanisms, and one of them is temperature-triggered gelation. Gelation can be induced by temperature change using the polymers like poloxamers. The temperature-triggered in situ gelling systems are prepared using thermosensitive polymers that exist as a liquid form below its low critical solution temperature (LCST), and a transition into gel form occurs when the environmental temperature reaches or is above the LCST [3]. These systems, particularly those with thermosensitive properties, have demonstrated their potential to enhance residence time and enable the controlled release of drugs for treating eye diseases and have shown their possible utilization in enhancing ocular absorption [4]. This capability arises from their ability to improve the bioadhesiveness of ophthalmic solutions [5]. In these systems, the gel is formed at the precorneal temperature to withstand dilution by lacrimal fluid, preventing rapid precorneal elimination of the administered drug [6]. A well-designed thermosensitive ocular in situ gel is advised to have a gelation temperature above room temperature and undergo a gel–sol transition at a precorneal temperature. This design helps avoid the need for refrigeration before instillation, as storing in a refrigerator could sometimes lead to eye irritation due to the cold nature [7]. 

The conventional liquid ophthalmic formulation is rapidly eliminated from the precorneal region upon administration because of the special anatomical structure of the eye, lacrimal secretion, and nasolacrimal drainage [8,9,10,11,12,13]. As a result of this situation, the short residence time in the precorneal area, combined with limited transcorneal absorption, leads to lower ocular bioavailability [14]. To enhance ocular bioavailability and prolong the duration of drug action, different ophthalmic carriers, such as viscous solutions, ointments, gels, or polymeric inserts, have been employed. The use of these vehicles led to varying degrees of increased contact time. However, because of issues such as blurred vision (e.g., with ointments) or lack of patient compliance (e.g., with inserts), their widespread acceptance has been limited [15]. Their rheological and mechanical properties are typically associated with drug release kinetics, bioadhesion, and the dosage form’s ability to endure mechanical stresses caused by bodily movements. 

Polyoxyethylene–polyoxypropylene copolymers, known as poloxamers, exhibit a distinctive characteristic of reversible thermal gelation. These copolymers have been extensively studied for their potential applications in ocular drug delivery systems [16,17]. Transparent and clear gels can be obtained using poloxamers [2]. The phase transition temperature strongly depends on poloxamer concentration [18], and P 188 is employed with poloxamer 407 to obtain the target sol–gel transition temperature [19]. At the same time, poloxamers can be used as a solubility-enhancing agent. P407 generates micelles and can solubilize hydrophobic molecules [2], and poloxamers may be considered as a potentially useful hydrophilic carrier in the preparation of SDs [20].

SDs of poorly water-soluble drugs in hydrophilic carrier matrices have been reported to improve their solubility and dissolution rate [21,22,23]. In this study, a melting method was used to prepare flurbiprofen–poloxamer 407 solid dispersions in a relatively easy, simple, quick, inexpensive, and reproducible manner.

Ocular inflammation can lead to serious consequences such as retinal opacification, cataracts, and even blindness. Keratitis, conjunctivitis, and uveitis can be counted among ocular inflammations [24,25]. Corneal blindness caused by ocular surface inflammation is the second most common cause of blindness in the world [26]. Since the possibility of injury and infection increases because the eye is exposed to the external environment for a long time and the eye surface is moist, effective treatments for inflammation are of great importance [27]. FB is a nonsteroidal anti-inflammatory drug that has been introduced in ocular therapy not only for the management of inflammatory diseases concerning ocular structures but also for use during eye surgery. It was selected as the model drug. 

The aims of this study include the following: 

(i) To increase the solubility of FB using the SD method; (ii) to develop ophthalmic thermogelling and bioadhesive systems containing FB-SDs; and (iii) to evaluate the effect of the bioadhesive polymer on the rheological, mechanical, and release properties of the formulations. 

## 2. Results and Discussion

### 2.1. Characterization of Solid Dispersions

#### 2.1.1. Solubility Studies

The solubility studies showed that the solubility of FB was increased using the solid dispersion method. The solubility of FB-SDs prepared with P407 was 12.95 ± 0.088 mg/mL, whereas the experimental water solubility of FB was determined as 0.039 ± 0.00127 mg/mL. Thus, it was seen that FB-SDs’ solubility in water increased 332-fold compared with FB. When the solid dispersion came in contact with water, the polymer particle hydrated rapidly (because of the high hydrophilic potency of P407) into the polymer solution, contributing to the increased wettability of drug particles [28]. On the other hand, it was observed that the solution of FB-SDs prepared using P188 became cloudy shortly after dissolution; therefore, the use of P188 was abandoned in the preparation of FB-SDs. 

#### 2.1.2. Determination of Drug Content

The drug content of the FB-SD samples (corresponding to 3 mg of FB) was found as 2.923 ± 0.006 mg with a recovery of 97.4 ± 0.2%. The UV spectrophotometric method was validated for linearity, specificity, accuracy, and precision according to ICH guidelines and was successfully used for the assay of FB in the determination of drug content and in vitro drug release studies. 

#### 2.1.3. Scanning Electron Microscopy (SEM)

**Figure 1:**
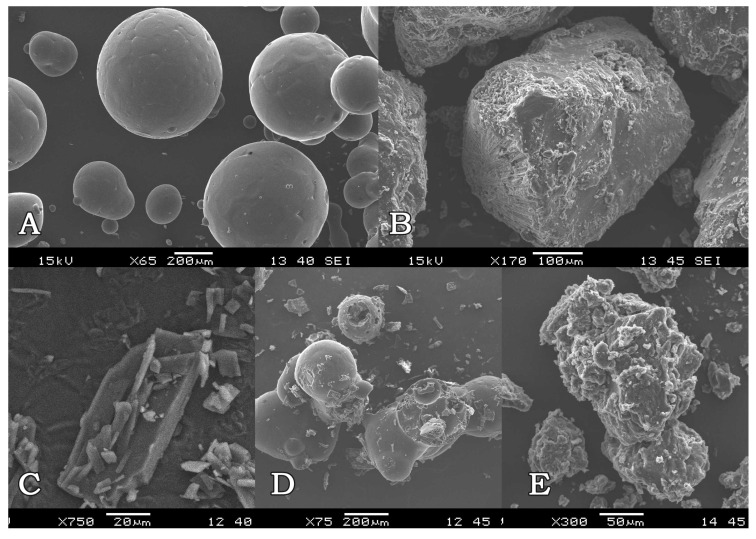
Scanning electron micrographs: (**A**) P407, (**B**) melted P407, (**C**) FB, (**D**) 1:10 (*w*/*w*) physical mixture, and (**E**) 1:10 (*w*/*w*) SD of FB:P407.

The SEM analysis provided information about the surface morphologies of solid dispersions and also showed the change in the surface properties of the active ingredient and polymer during the preparation of formulations. When the electron microscope images were examined, it was seen that FB consisted of a flat-surfaced rectangular crystal structure, while P407 consisted of flat-surfaced spherical particles, and there were changes on the surface of melted P407. Regarding the microscope images of the SDs, it was observed that particles with a uniform and rough surface were formed, and the surface properties of P407 and FB changed during the melting and solidification processes in the preparation of SD, while in PM, individual surface properties of FB and P407 were maintained (Figure 1). Based on these data, it was thought that FB, a slightly water-soluble substance, and the hydrophilic carrier system (P407) mixed homogeneously, showing a mass with a wrinkled surface. 

#### 2.1.4. Differential Scanning Calorimetry (DSC)

**Figure 2:**
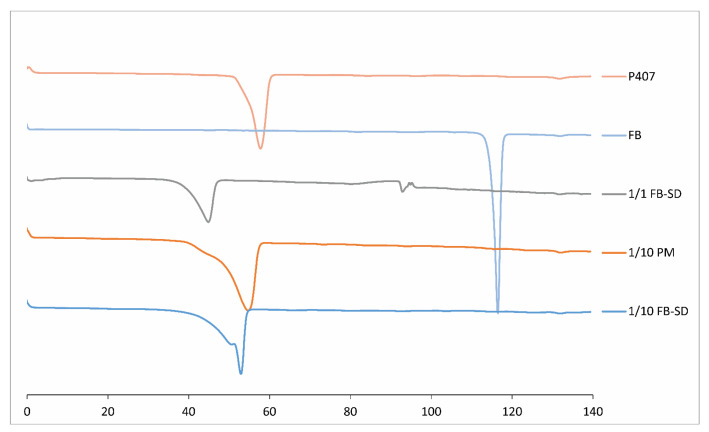
DSC thermograms of FB, P407, and PM in the ratio of 1/10 (*w*/*w*) FB:P407 and FB-SDs in the ratios of 1/1 and 1/10 (*w*/*w*) FB:P407.

By measuring the changes in the melting degrees and energies of the substances with DSC, information was obtained about the thermal interactions between the substances. When the DSC thermograms were examined, it was observed that FB gave a sharp endothermic peak at 116.53 °C, corresponding to its melting point, and the poloxamer gave a peak at 57.86 °C [29]. The thermograms showed that in the FB-SDs with the ratio of 1/1, the peaks of FB and P407 were shifted to 92.81 °C and 44.88 °C, respectively (Figure 2). Since no active substance peak was observed in the DSC thermograms of the 1/10 (*w*/*w*) PM and SDs, it was thought that there was an interaction between the active substance and polymer, and FB was molecularly dispersed or existed in the amorphous form. 

#### 2.1.5. Thermogravimetric Analysis (TGA)

**Figure 3:**
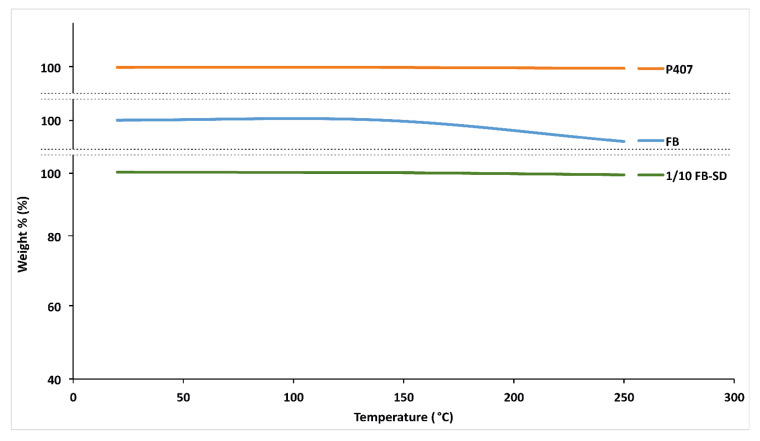
TGA thermograms of FB, P407, and FB-SDs in the ratio of 1/10 (*w*/*w*) FB:P407.

TGA analysis is based on the measurement of weight changes resulting from reactions such as the decomposition and degradation of substances. In this study, thermogravimetric analyses were conducted between 0 and 250 °C to demonstrate that FB, P407, and SDs did not undergo degradation at 140 °C, which is the preparation temperature of SDs. The degradation was found to be less than 1% and, based on the literature, this value indicates that FB, P407, and SDs are thermally stable at 140 °C (Figure 3) [30].

#### 2.1.6. X-ray Powder Diffraction Studies

**Figure 4:**
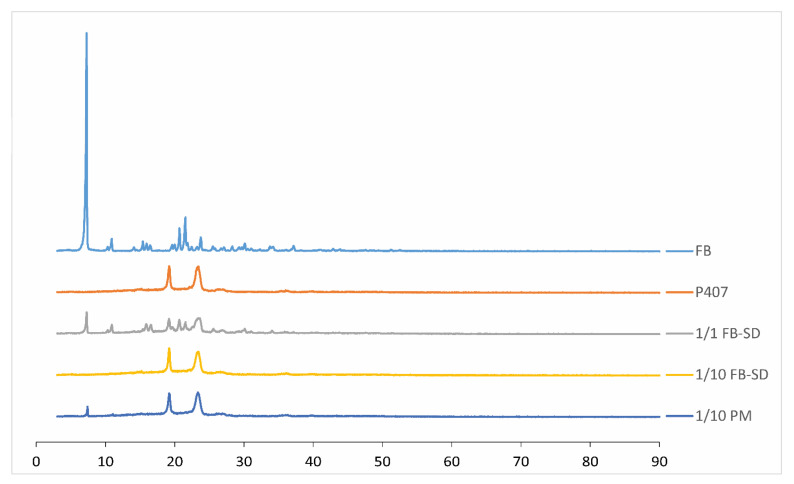
X-ray diffraction patterns of FB, P407, and the physical mixture in the ratio of 1/10 (*w*/*w*) FB:P407 and FB-SDs in the ratios of 1/1 and 1/10 (*w*/*w*) FB:P407.

To support the results obtained from DSC, X-ray diffraction analysis was performed to determine whether FB existed in a crystalline form within the SDs. Since the presence of FB in a crystalline and undissolved form within the formulation could lead to eye irritation, this analysis aimed to determine the status of FB in the formulation (crystalline, amorphous, or molecularly dissolved). Upon examining the obtained X-ray diffraction patterns, it was observed that the active substance existed in crystalline form in the physical mixture prepared in the ratio of 1/10 (*w*/*w*). However, in the SDs, the sharp peaks indicating the crystalline structure of FB were lost, suggesting a transition to an amorphous form or molecular dispersion. In the SDs prepared at a 1/1 (*w*/*w*) ratio, it was observed that FB retained its crystalline structure (Figure 4). 

#### 2.1.7. Stability Studies

Stability studies were carried out for 2 months for the characterization of solid dispersions, and it was found that solid dispersions could be stored at room temperature and in the refrigerator during this period without decomposition (Table 1).

### 2.2. Characterization of Thermosensitive and Bioadhesive Ophthalmic Formulations

#### 2.2.1. Gelation Temperature and Gelation Time

The phase transition temperature, i.e., the temperature at which the liquid phase makes a transition to a gel, is obviously an important parameter for in situ gelling systems. The suitable gelation temperature for the in situ gelling ophthalmic formulations was stated to be between 32 and 35 °C in the literature [14,19,31,32,33,34,35], and it was suggested that the gelation temperature should be between 25 °C, the average ambient temperature, and 35 °C, the temperature of the eye surface. Thus, the ophthalmic formulation could be in liquid form at average ambient temperature and form a gel phase instantly on the ocular surface [36].

The gelation temperature of the ophthalmic formulations containing FB-SDs was dependent on the concentration of P407 and P188 and also the bioadhesive polymers. Therefore, various mixings of poloxamers containing FB-SDs were prepared firstly to obtain optimum gelation temperature, and their gelation temperatures were determined using the first method. It was seen that the increase in the amount of P 188 decreased the gelation temperature, and the decrease in the concentration of P 407 from 24% to 23% resulted in an increase in the gelation temperature from 33.2 to 36 °C. These results fit with our previously obtained results [37]. The gelation of poloxamer solutions, which varies with temperature, could be elucidated by a change in configuration [38,39]. The molecules of a poloxamer demonstrate a neatly arranged zigzag formation. As the temperature increases, the zigzag configuration of a poloxamer can transform into a close-packed meander arrangement, resulting in a more close-packed and more viscous gel [40]. To elaborate further, a poloxamer is a type of block copolymer, composed of units of poly(oxyethylene) (PEO) and poly(oxypropylene) (PPO) [41]. The gel formation in poloxamer block copolymers is primarily due to the structured arrangement of micelles [42]. These spherical micelles feature a core of dehydrated PPO encased in a shell of swollen, hydrated PEO chains [43]. An increase in temperature results in dehydration and conformational changes within the regions of hydrophobic chains, enhancing the friction between chains and the entanglement of the polymer network [41,44]. A greater amount of unbound water is present in the hydrophilic regions of the gel [45]; thus, the external PEO chains interpenetrate in the gel extensively. At this stage, gelation occurs, and the micelles remain visibly intact and orderly packed, a process often referred to as “hard-sphere crystallization” [46]. Variations in the hydrophilic and hydrophobic segments of the P407 and P188 compositions in a mixed solution also impact the gelation temperature of their mixtures. The influence of low concentrations of P188 aligns well with the findings of Yuan et al. [47], who observed a rising gelation temperature trend when adding a low to intermediate amount of P188 to the blend. Solutions with a higher content of hydrophobic PPO exhibit lower gelation temperatures, whereas those rich in hydrophilic PEO display high gelation temperatures. P188, being more hydrophilic than P407, consists of a greater PEO/PPO ratio compared with P407. The addition of a small amount of P188 primarily modifies the PEO ratio within the polymer blend, leading to an increase in the gelation temperature. Nonetheless, a relatively high concentration of P188 could initiate the micellization of P188 within the mixture. Beyond the changes in the PEO/PPO ratio in the mixed solution, an increase in P188 levels is believed to facilitate the formation of P188 micelles, aiding in the gel structure. Consequently, the gelation temperature decreased with an additional increase in P188 content [42]. Among the eight preformulations of the P407/P188 mixtures, 15/26.5% containing FB-SDs equivalent to 0.03% FB, which gels at 33.9 ± 0.71 °C, referring to the temperature range of 32–35 °C, were selected as the suitable formulation for preparing in situ gelling ophthalmic formulations (Table 2).

The effect of the addition of bioadhesive polymers in different concentrations into the selected formulation P407/P188 (15/26.5%) containing FB-SDs equivalent to 0.03% FB on the gelation temperature was also determined using oscillation measurements.

**Figure 5:**
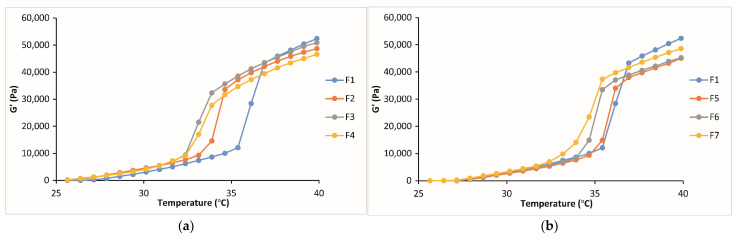
G′ vs. temperature curves of the formulations containing (**a**) CP and (**b**) CMC in different concentrations.

The sol–gel transition temperatures, in other words, gelation temperatures, correspond to the temperature characterized by a drastic change in the rheological behavior and elastic modulus. The elastic modulus G′ is a measure of the energy stored and recovered per cycle of deformation and reflects the solid-like component of elastic behavior. The elasticity modulus is low at the solution stage but increases drastically at the temperature required to form a gel [48]. According to the oscillation study, G′ vs. temperature values for the formulations containing 0.2, 0.4, and 0.6% CP (F2, F3, F4) indicated that they start to gelify between the temperatures of 32 and 33 °C, whereas the formulations containing 0.2, 0.4, and 0.6% CMC (F5, F6, F7) start to gelify between the temperatures of 33 and 34 °C. The formulation P407/P188 (15/26.5%) containing FB-SDs (F1) showed the maximum gelation temperature among the formulations, and it was seen that the addition of bioadhesive polymers decreased the gelation temperature. The gelation temperatures were also affected by the concentrations of the bioadhesive polymers. It was seen that the decrease in gelation temperature was higher in the formulations prepared with CP than in the formulations prepared with CMC (Table 3, Figure 5). The obtained results are compatible with the current literature [40,49]. Mucoadhesive polymers exhibit a reduction in gelation temperature, attributed to their capability to bind to the polyoxyethylene chains within poloxamer molecules. This situation facilitates dehydration, thereby enhancing the entanglement of the adjant and significantly elevating intermolecular hydrogen bonding. Consequently, this phenomenon of molecules culminates in gelation at lower temperatures [49,50,51]. Regarding the results of the two different methods, the gelation temperatures were found to be very similar (Table 3).

The gelation time is another factor for ophthalmic administration of drugs; it should be as short as possible to avoid a quick removal of the polymer solution by lachrymal fluid. After administration of an ophthalmic formulation, patients should hold the head back for a few minutes, or keep lying down. The gelation time is considered to be the waiting time in the lying position to avoid the drug movement from the cul-de-sac by the tear fluid and blinking before gelation occurs [19,34,52]. 

**Figure 6:**
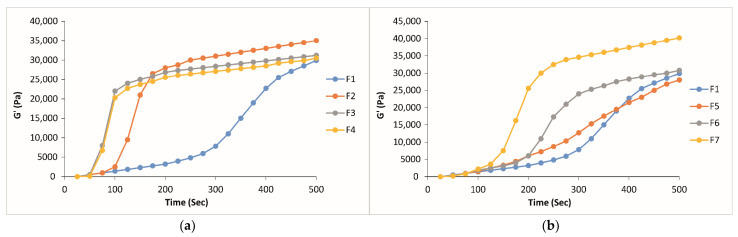
G′ vs. time curves of the formulations containing (**a**) CP and (**b**) CMC in different concentrations.

Figure 6 shows G′ profiles as a function of time for the formulations containing CP or CMC in different concentrations respectively. It can be seen that CP-containing formulations gel in a shorter time because of the increase in bioadhesive polymer concentration, as evidenced by high G′ values between 71 and 120 s (Table 3). On the contrary, the formulations containing CMC are characterized by a slower increase in G′, meaning a slower gelation of the sample.

Regarding the results of the oscillation study, it was concluded that the addition of bioadhesive polymers shortened the gelation time. Additionally, the gelation time was decreased with increasing bioadhesive polymer concentration, and the formulations prepared with CP had a shorter gelation time.

The osmololarity of all thermosensitive and bioadhesive ophthalmic formulations, as determined by an osmometer was found to be between 300 and 320 mosm/L. These values fit with the literature [53,54].

#### 2.2.2. Rheological Evaluation of Thermosensitive and Bioadhesive Ophthalmic Formulations

**Figure 7:**
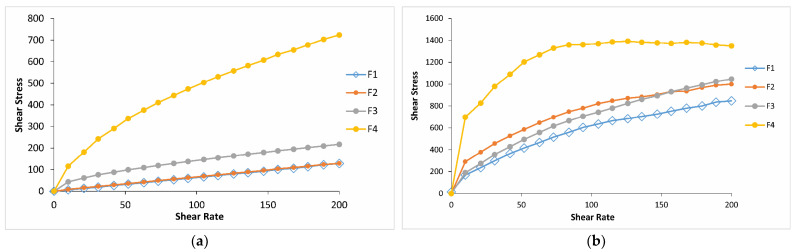
Shear stress vs. shear rate flow curves of the formulations containing CP in different concentrations (**a**) at 25 °C and (**b**) at 34 °C.

**Figure 8:**
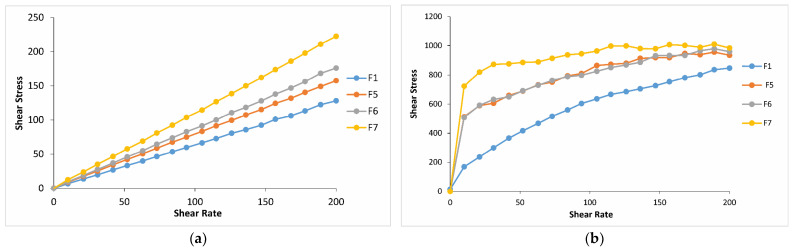
Shear stress vs. shear rate flow curves of the formulations containing CMC in different concentrations (**a**) at 25 °C and (**b**) at 34 °C.

Rheological studies are an important criterion in predicting the in vivo behavior of formulations when applied to the eye. Since the flow property of a viscous polymer solution affects its residence time and behavior in the precorneal area, rheological properties provide insight for choosing the most appropriate system in terms of viscoelasticity [55]. A critical attribute that determines the dosing accuracy, retention time, and drug release from in situ forming ophthalmic gels is formulation rheological behavior. The systems with suitable rheological properties would withstand high shear rates and tear dilution, thus preventing the drainage of a drug from the absorption site [33]. In situ thermo-gelling systems are free-flowing liquid at room temperature (25 °C) for easy application; however, they undergo a sol–gel transition at physiological temperature to be resistant to the shear forces in the conjunctival cul-de-sac [36,56]. The thermoreversible properties of the formulations were evaluated by rheological parameters, such as the shear stress changes upon shear rates, and the sol–gel transition temperature. The shear stress changes upon shear rates have been analyzed to specify the rheological behavior of the formulations (Newtonian or non-Newtonian) both at physiological and non-physiological conditions. Regarding the obtained results, it was seen that for all the formulations except for F3 and F4 at non-physiological conditions, the shear stress increased linearly with an increase in shear rate, demonstrating a Newtonion flow behavior [57,58]. All the formulations at physiological conditions and formulations F3 and F4 containing 0.4 and 0.6% CP, respectively, at both conditions showed non-Newtonion pseudoplastic flow. Meanwhile, it should not be overlooked that formulations F3 and F4, exhibiting pseudoplastic flow at room temperature, may be more challenging to remove from their packaging, leading to application difficulties. For all the formulations studied, the shear stresses at 34 °C were higher than those at 25 °C. For instance, at a shear rate of 100 s^−1^, the shear stresses of the formulations containing 0, 0.2, 0.4, and 0.6% CP (F1, F2, F3, and F4) at physiological conditions were approximately 8.5, 11.5, 6, and 3 times greater than those at non-physiological conditions, respectively (Figure 7). A similar situation was observed in the formulations containing CMC at the same ratios (F1, F5, F6, and F7), with approximately 8.5, 10, 9, and 7.5 times greater increases, respectively (Figure 8). These results are important for suggesting the occurrence of phase transition between these two conditions for both systems. Moreover, it was observed that in both temperatures, the increase in CP or CMC concentrations in the formulations led to a proportional increase in shear stresses. The increase in shear stress observed in poloxamer solutions transitioning from non-physiological to physiological conditions was influenced by temperature and can be elucidated by the structural characteristics of the poloxamer, which is a triblock copolymer. In poloxamers, PEO is primarily hydrophilic, while PPO exhibits hydrophilicity at low temperatures and shifts towards increased hydrophobicity at higher temperatures. After the combination of PEO and PPO blocks, the emergence of amphiphilic characteristics and aggregation phenomena can be anticipated at elevated temperatures [59]. In other words, if the polymer concentration and the characteristic temperature surpass a critical point, micelles are formed by this triblock copolymer [60,61]. The creation of micelles can elevate the viscosity of vehicles and lead to the sol–gel transition at elevated temperatures [62,63].

**Figure 9:**
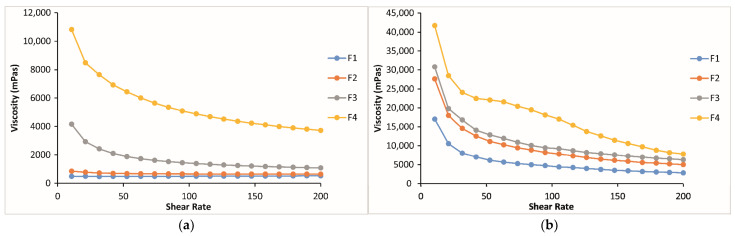
Viscosity vs. shear rate flow curves of the formulations containing CP in different concentrations (**a**) at 25 °C and (**b**) at 34 °C.

**Figure 10:**
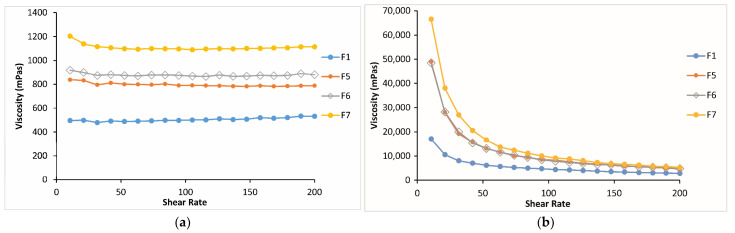
Viscosity vs. shear rate flow curves of the formulations containing CMC in different concentrations (**a**) at 25 °C and (**b**) at 34 °C.

It was shown in the literature that dilution by artificial tear fluid had a great influence on shear stress under physiological conditions, and it was suggested that the in situ gelling ophthalmic formulation composed of P407/P408 and 0.2% CP1342 showing a Newtonian flow behavior under physiological conditions cannot turn into a hard gel because of its lower concentration and dilution by artificial tear fluid. The authors determined that this formulation in in vivo use may not have enough strength to withstand the turnover and dilution of the lacrimal fluid, which may lead to a short precorneal residence time, although it has an excellent mucoadhesive property [14]. This problem can be overcome by using in situ-gelling ophthalmic formulations that exhibit reversible phase transitions with increased viscosity values and pseudoplastic behavior, thus increasing the precorneal residence of the formulation and enhancing ocular bioavailability [34]. The results of our study indicated that all the formulations showing pseudoplastic flow have increased viscosity values at the physiological condition, according to the viscosity vs. shear rate flow curves, and the viscosity values increased with the increase in bioadhesive polymer concentration in the formulations. Only the formulations having 0.2 and 0.4% CP (F2 and F3) or CMC (F5 and F6) showed almost the same viscosity, according to the viscosity vs. shear rate flow curves (Figure 9 and Figure 10).

On the other hand, the viscous ophthalmic solutions showing pseudoplastic rheological behavior offer less resistance to the eyelids during blinking and, therefore are expected to be more comfortable in the eye than Newtonian solutions [64,65].

### 2.3. Mechanical Properties of Thermosensitive and Bioadhesive Ophthalmic Formulations

Textural analyses offer insights into the mechanical characteristics of samples, including hardness, compressibility, and adhesiveness. These attributes can be directly linked to sensory parameters in vivo, thus proving invaluable in crafting a product with desirable features that enhance patient acceptability and compliance [36,66].

The results of the texture profile analysis at 25 °C and 34 °C showed that the type and concentration of the bioadhesive polymers influence the mechanical properties of the formulations. It was seen that all formulations studied at 25 °C have low hardness, compressibility, and adhesiveness properties.

It is known that products with low hardness and compressibility properties are easily removable from packaging and, because of this, they are administration-friendly, potentially leading to a positive perception by the patient [36,66].

**Figure 11:**
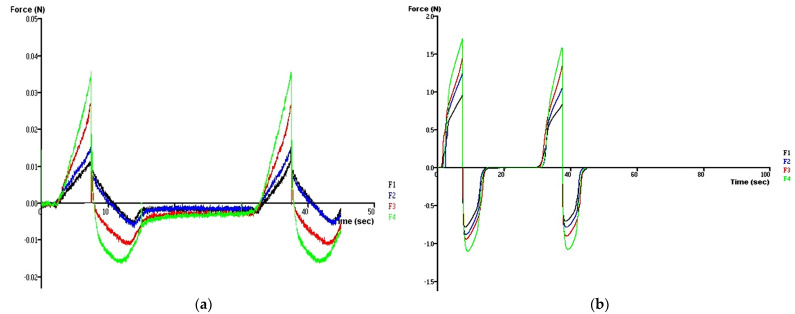
Mechanical properties of formulations containing CP in different concentrations, determined using texture analysis at 25 °C (**a**) and 34 °C (**b**).

**Figure 12:**
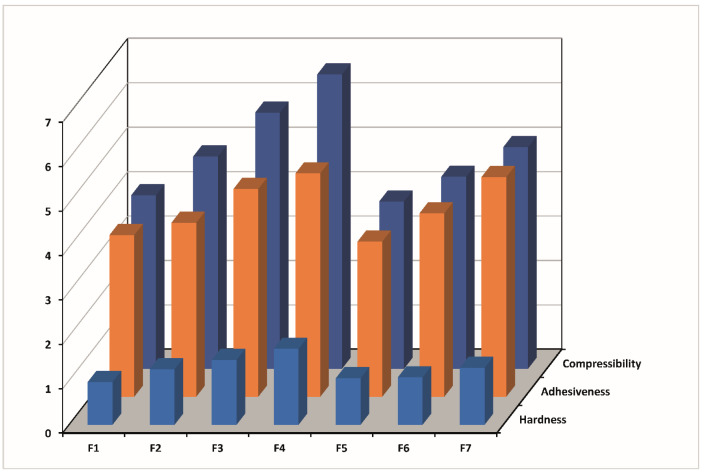
Mechanical properties (hardness, adhesiveness, and compressibility) of formulations determined using texture profile analysis at 34 °C.

On the other hand, according to the time–force plots, it was observed that formulation F1 has lower mechanical properties than the other formulations. In addition, the hardness, compressibility, and adhesiveness values of the formulations increased with the addition of CP and with increasing the concentration of CP at 25 and 34 °C (Table 4 and Figure 11 and Figure 12).

The formulations prepared with CMC showed the same results at 34 °C (Table 4, Figure 12). However, the increase in CMC concentration had no significant effect on the mechanical properties of formulations prepared at 25 °C (Table 4). 

At 34 °C, the formulation has already been administered and is a gel in the eye. In this scenario, it is preferable for the formulation to have a specific level of hardness to resist drainage, as a formulation that easily flows would quickly dilute in tears and drain away. Some authors have explored the correlation between hardness (strength) and formulation retention time [66,67]. Therefore, formulations F3 and F4 containing 0.4 and 0.6% CP, respectively, are expected to hold for a prolonged time on the corneal surface before drainage. Compressibility can also be associated with the effort required to distribute the product evenly over a specific surface. Following the phase transition at eye temperature, it is preferable for the gel to establish a uniform layer on the corneal surface, thus preventing patient discomfort and blurred vision, while aiding the diffusion of the drug. Therefore, it is desirable to have a low compressibility value. Our results showed that the compressibility values at 34 °C increased with increasing bioadhesive polymer concentration. The compressibility values of the formulations containing CMC were lower in the formulations containing CP, and the lowest compressibility value was obtained in the formulation containing 0.2% CMC (F5) (Table 4, Figure 12). Adhesiveness is commonly defined as the work necessary to overcome the attractive forces between the surface of the sample, and the surface of the probe [68] and is a property related to mucoadhesion [69]. It is a preferred characteristic, as a higher adhesiveness value may indicate stronger adherence to the tissue surface, thereby enhancing the retention time [70] and improving clinical efficacy [71]. In this study, the addition of CP or CMC at the concentration of 0.6% (F4 and F7) significantly increased formulation adhesiveness compared with the formulation without bioadhesive polymer (F1) at 34 °C (Table 4, Figure 12). However, the formulation containing 0.6% CP (F4) showed the maximum adhesiveness among all formulations. 

Earlier research on CP polymers clearly suggested that the presence of carboxyl groups is the determining factor for bioadhesion [72]. CP, characterized by a notably high percentage (58–68%) of carboxylic groups, gradually engages in hydrogen bonding with sugar residues within oligosaccharide chains present in the mucous membrane. This interaction leads to the formation of a reinforced network between the polymer and the mucous membrane. Consequently, CP, with its high density of available hydrogen bonding groups, can establish a stronger interaction with mucin glycoproteins. Additionally, CP may adopt a more favorable macromolecular conformation, enhancing the accessibility of its functional groups for hydrogen bonding. It is hypothesized that the higher mucoadhesive strength of the delivery system could result in prolonged retention and increased absorption across mucosal tissues [73].

The strong effect of CP on the mechanical properties of the formulations is thought to be due to its carboxyl groups, which can form strong bonds with the cross-linked reticular poloxamer gel by positioning its molecules between the gel [40].

### 2.4. In Vitro Drug Release from Thermosensitive and Bioadhesive Ophthalmic Formulations

**Figure 13:**
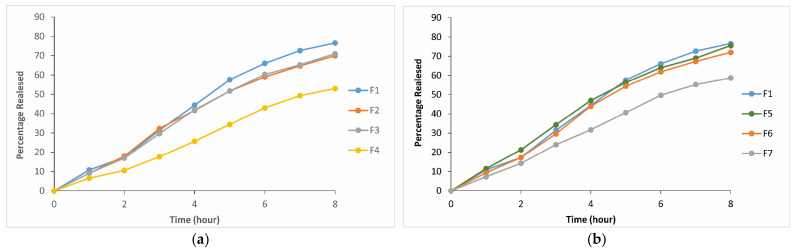
Effect of (**a**) CP and (**b**) CMC in different concentrations on the release of FB from thermosensitive and bioadhesive ophthalmic formulations.

After the selection of the formulation F1 (P407/P188 (15/26.5%)) containing FB-SDs equivalent to 0.03% FB with suitable gelation temperature (33.9 °C), CP or CMC was added to this formulation as a mucoadhesive polymer in different ratios to test their effects on the release rate of FB. The release of FB was variously affected by the mucoadhesive polymers and their concentration in the formulation. Based on the obtained results of the in vitro drug release studies, CP and CMC have no effect on the release rate below the concentration of 0.6%. On the other hand, in the formulation without bioadhesive polymer (F1), 76.6% of FB was released within 8 h, while in the formulations containing 0.6% CP or CMC (F4 and F7) and 53% and 58.7% of FB were released within 8 h, respectively. It was determined that the reason for the decrease in the release rate with the addition of CP is the increase in gel hardness with the increase in CP concentration. A higher gel hardness means stronger viscosity and a more compact structure of poloxamer molecules in formulations. CP, which enhances gel hardness and decreases gelation temperature, could distort or squeeze the diffusion channels, delaying the release process. This result agrees with the literature (Table 3 and Table 4, Figure 12) [74]. In light of these data, it was determined that formulations F4 and F7 showed low-release properties (Figure 13).

Since some mechanical properties of formulation F1, such as hardness and adhesiveness, are lower than other formulations, it was concluded that formulation F1 did not show the desired properties to apply to the eyes.

### 2.5. Kinetic Evaluations

When the formulations were evaluated kinetically, determined coefficients (r^2^) and the residuals were calculated. Accordingly, first-order kinetic and Hixson–Crowell kinetic models with determination coefficients (r^2^) closest to 1 were chosen as models explaining the release kinetics of the formulations (Table 5). In the first-order model, drug activity within the reservoir was assumed to decline exponentially, and the rate of drug release was proportional to the residual activity. The Hixson–Crowell model was developed to describe the release from dosage forms that show dissolution rate limitation and do not dramatically change during the release process [75]. 

When n exponent results were evaluated to understand the release mechanisms of FB from thermosensitive and bioadhesive ophthalmic formulations, it was seen that the n values of the formulations are greater than 1, except for formulation F5, suggesting that FB might be released from the ophthalmic formulation by super case II type diffusion (Table 6). This result describes drug diffusion from the gels showing plastic or pseudoplastic flow. First-order and Hixon Crowell kinetics chosen as models explaining release kinetics of the formulations fit with the results of Peppas equation. Since formulation F5 was prepared with a small amount of CMC, such as 0.2%, the n value was found to be below 1 and showed a release fitting with non-Fickian diffusion, which represents first-order kinetics resulting from swelling and relaxation in the polymer structure. The insufficient drug release in the F4 formulation led to a deviation towards zero-order kinetics in the kinetic modeling (Table 5).

## 3. Conclusions

This study described the design and development of bioadhesive and thermosensitive in situ gelling ophthalmic formulations containing FB-SDs. Ophthalmic formulations were successfully prepared using P407, P188, and two different types of bioadhesive polymers in various concentrations. According to the release and rheological and mechanical properties, it was concluded that the formulations F2, F5, and F6 containing P407/P188/CP (15/26.5/0.2%), P407/P188/CMC (15/26.5/0.2%), and P407/P188/CMC (15/26.5/0.4%), respectively could be promising formulations as an anti-inflammatory ophthalmic dosage form of FB for effective therapy because of their suitable gelation temperature, adequate release characteristics, and suitable mechanical properties. 

According to their mechanical properties, the following conclusions were drawn:

They will remain resistant to tear drainage, they will be easier to adhere to the eye and their retention time in the eye will be longer, they will maintain their gel shape for a long time after gelling in the eye, and they will gel in a homogeneous layer on the eye surface and they will not cause any discomfort to the patient in the form of blurred vision during drug diffusion. 

They will be easier to remove from their packages and can be applied easily in terms of both their mechanical properties and rheological properties at 25 °C. 

Since they show pseudoplastic flow properties at 34 °C, their viscosity will increase following application to the eye, thus preventing their removal from the eye by diluting with tears and increasing the retention time in the eye.

## 4. Materials and Methods

### 4.1. Materials

P407 and P188 were gifted from BASF (Ludwigshafen, Germany). CP was kindly supplied by Lubrizol (Cleveland, OH, USA). CMC was purchased from Sigma (St Louis, MO, USA), and FB was kindly supplied by Sanovel (İstanbul, Turkey). All other chemicals were used at analytical grade.

### 4.2. Preparation of Solid Dispersions

FB-SDs with a 1/10 (*w*/*w*) flurbiprofen:poloxamer ratio were prepared by the fusion method. According to this technique, the required amount of P407 or P188 and FB were weighed accurately and heated to 140 °C with a constant stirring rate until it formed a transparent melt. The melt was then poured onto aluminum foil and allowed to solidify at 4 °C. The solid mass was powdered and mixed uniformly in a mortar, and FB-SDs were so obtained [20,76].

### 4.3. Characterization of Solid Dispersions

#### 4.3.1. Solubility Studies

Excess amounts of FB or FB-SDs were added to 10 mL HPLC-grade water. The samples were mixed under magnetic stirring (300 rpm) at 25 °C in a temperature-controlled water bath until equilibrium was achieved. The samples were subsequently filtered through a 0.45 µm membrane filter, and after suitable dilutions, they were analyzed using a UV spectrophotometer at 248 nm. The solubility experiments were conducted in triplicate, and the mean ± SD was reported [77].

#### 4.3.2. Determination of Drug Content

An accurately weighed amount of FB-SD samples (corresponding to 3 mg of FB) were solubilized in 10 mL of HPLC-grade water, and after suitable dilutions, they were analyzed spectrophotometrically at 248 nm. Experiments were performed in triplicate, and the drug content% was calculated. The UV spectrophotometric method was validated in our laboratory in accordance with the Q2(R1) ICH Guideline [78]. 

#### 4.3.3. Scanning Electron Microscopy (SEM)

The surface morphology of FB, P407, melt P407, physical mixture (PM) of FB and P407 in the ratio of 1/10 (*w*/*w*), and FB-SDs was examined using a scanning electron microscope (JSM–6060 JEOL Ltd., Tokyo, Japan). The samples were fixed on a brass stub using double-sided adhesive tape and made electrically conductive by coating with platinum and scanned at an accelerating voltage of 15 kV. 

#### 4.3.4. Differential Scanning Calorimetry (DSC)

Thermal analysis was performed on a Perkin Elmer DSC 8000 differential scanning calorimeter (Perkin Elmer Inc., Shelton, CT, USA) for the samples of FB, P407, PM of FB, and P407 in the ratio of 1/10 (*w*/*w*) and FB-SDs in the ratios of 1/1 and 1/10. The accurately weighed sample was placed in an aluminum pan. An empty aluminum pan was used as a reference. The experiment was carried out in nitrogen atmosphere at a scanning rate of 10 °C/min over a temperature range of 0 to 140 °C.

#### 4.3.5. Thermogravimetric Analysis (TGA)

The thermal stability of FB, P407, and FB-SDs was evaluated by TGA. A sample of approximately 5 mg was weighed into an aluminum pan and placed into the furnace of a TGA instrument (TGA 4000, Perkin Elmer Inc. Shelton, CT, USA). The thermal stability of the samples was monitored from 0 to 250 °C employing a heating rate of 10 °C/min. 

#### 4.3.6. X-ray Powder Diffraction Studies

Diffraction patterns of the samples (FB, P407, PM of FB, and P407 in the ratio of 1/10 (*w*/*w*) and FB-SDs in the ratios of 1/1 and 1/10 (*w*/*w*)) were recorded with a X-ray diffractometer for powders (Rigaku Co., Tokyo, Japan). A voltage of 40 kV for the generator was used, with Cu as the tube anode material. The solids were exposed to Cu-Kα radiation, over a range of 2θ angles from 3° to 70°, at an angular speed of 10° per minute.

#### 4.3.7. Stability Studies

FB-SDs equivalent to 15 mg FB were dissolved in a volumetric flask with 0.6 mL ethanol and then completed to 25 mL with phosphate buffer pH 7.4. Samples prepared from this solution with appropriate dilutions were stored separately at 25 ± 2 °C under a relative humidity of 60 ± 5% in a chamber for stability testing, in the refrigerator (5 ± 3 °C) and in the deep freezer (−20 ± 5 °C), for two months (according to ICH guideline Q1A (R2) [79]. The absorbances of the samples were recorded initially and at certain time intervals spectrophotometrically at 248 nm.

### 4.4. Preformulation Studies of Thermosensitive Ophthalmic Formulations

Preformulation studies were carried out to determine the gelation temperature, which is an important factor in preparing ophthalmic formulations. Gelation temperature is defined as the temperature at which the liquid phase makes a transition to a gel. Gelation temperature for the in situ gelling ophthalmic formulations is stated to be between 32 and 35 °C in the literature, and in this study, 34 °C from this range was chosen as the optimum gelation temperature for the ophthalmic formulations [80]. For this reason, gelation temperature was determined firstly by mixing different ratios of P407 (15–24%) and P188 (5–27%) and then by adding solid dispersions containing 0.03% of FB to this mixture (Table 2). Regarding the obtained results, the optimum ratio of the P407 and P188 mixture, which was used for the preparation of ophthalmic formulations, was selected.

### 4.5. Preparation of Thermosensitive and Bioadhesive Ophthalmic Formulations

FB-SDs containing the required amount of FB (0.03%) were completely dissolved in HPLC-grade water with continuous agitation at room temperature, and bioadhesive polymers with different concentrations were added and then cooled down to 4 °C. The mixture of P407 and P188 was then slowly added to the solution with continuous agitation. The liquid solution was left at 4 °C overnight until a clear solution was obtained. The pH was adjusted to 7.4 ± 0.5 using 0.01 M NaOH solution. Finally, the volume was increased to 5 mL with HPLC-grade water at pH 7.4 [34,81,82]. The osmololarity of the formulation was determined by an osmometer (Semi-Micro-Osmometer, Knauer, Berlin-Zehlendorf, Germany).

### 4.6. Characterization of Thermosensitive and Bioadhesive Ophthalmic Formulations

#### 4.6.1. Measurement of Gelation Temperature

Two different methods were used to determine the gelation temperature of the ophthalmic formulations. For the first method, a 20 mL transparent vial containing a magnetic bar and 5 mL of ophthalmic solution was placed in a low-temperature thermostat water bath. A digital thermosensor (Alla, Chemille, France) connected to a thermistor was immersed in the ophthalmic solution, and it was heated at a constant rate (1 °C/min) with constant stirring (130 rpm). The gelation temperature was determined as the temperature registered on the thermistor when the magnetic bar stopped moving because of gelation [40,83]. The second method was oscillation measurements described in Section 4.6.2, which was used to determine gelation temperature and gelation time [84].

#### 4.6.2. Rheological Evaluation of Thermosensitive and Bioadhesive Ophthalmic Formulations

The rheological analysis was carried out by means of a rotational rheometer (Haake Mars Rheometer, Thermo Fisher Scientific, Karlsruhe, Germany), equipped with a cone–plate combination (the cone had a 35 mm diameter and a 2 ° angle) as a measuring system. The shear stress of the formulations was determined at different shear rates at 25 ± 0.1 and 34 ± 0.1 °C, respectively. A typical run comprised changing the shear rate from 0 to 200 s^−1^ at a controlled ramp speed (keeping a period of 6 s at each shear rate). Evaluations were conducted in triplicate [14,57]. According to the obtained graphs of shear stress vs. the shear rate and viscosity vs. the shear rate, the flow type of ophthalmic formulations was determined.

In particular, the formulations were subjected to oscillation measurements, which apply a constant shear stress value (chosen in the linear viscoelastic region, previously determined) and measure the viscoelastic response of the formulation expressed by the storage (G′) and loss (G″) moduli, which are characteristic of the stored elastic energy and the viscous dissipated energy, respectively. It is known that the elastic and viscous moduli values and their ratios provide information about rheological characteristics and comparison of various viscoelastic formulations [85]. The oscillation measurements were performed as follows:

- At a constant frequency value (1 Hz) and at temperature values ranging between 25 and 40 °C to evaluate the gelation temperature of the samples (Method 2).
- At constant temperature (34 °C) and frequency (1 Hz) values and at increasing times to determine the gelation time of the sample at the physiological temperature.

The evaluations were conducted in triplicate [84].

#### 4.6.3. Mechanical Properties of Thermosensitive and Bioadhesive Ophthalmic Formulations

Texture profile analysis was performed using a texture analyzer (Model TA-TX Plus, Stable Micro System Ltd., Godalming, Surrey, UK) in TPA mode. The formulations were transferred into a 10 mL beaker and packed to a fixed height, and the temperature of each sample was calibrated to 25 ± 0.5 °C and 34 ± 0.5 °C. The analytical probe was compressed twice into each sample to a depth of 15 mm at a rate of 2.0 mms^−1^. A delay period of 15 s was allowed between the end of the first compression and the beginning of the second compression. All tests were performed at least in triplicate. From the resultant force–time plot, several mechanical parameters may be defined including, hardness, compressibility, and adhesiveness [86,87].

#### 4.6.4. In Vitro Drug Release from Thermosensitive and Bioadhesive Ophthalmic Formulations

The in vitro drug release of FB from the ophthalmic thermosensitive and bioadhesive formulations was evaluated by a dialysis method. The ophthalmic formulations (5 mL) were placed into a pre-swollen dialysis bag with a molecular weight cut of 6–8 kD (spectra/Por 1) and immersed into a beaker containing 200 mL phosphate buffer pH 7.4, which was maintained at 34 ± 0.5 °C under constant magnetic stirring at 300 rpm. A sample (3 mL) was withdrawn from the dissolution medium at regular intervals, which were then assayed spectrophotometrically at 248 nm. After spectrophotometric analysis, the samples were poured into a dissolution medium [88]. The experiments were performed in triplicate.

#### 4.6.5. Kinetic Evaluations

The results thus obtained were evaluated kinetically by zero-order, first-order, Higuchi, and Hixson–Crowell equations. The determination coefficients (*r^2^*) and the residuals were calculated by means of a computer program [89]. The mechanism of release of FB from the ophthalmic formulations was analyzed using Equations (1) and (2), where *Mt/M* is the fraction of released drug at time *t*, *k* is a release characteristic constant of the ophthalmic formulation, and *n* is a release exponent indicative of the release mechanism. As the *k* value becomes higher, the drug is released faster. The *n* value of 1 corresponds to zero-order release kinetics, 0.5 < *n* < 1 means a non-Fickian release model, and *n* = 0.5 indicates Fickian diffusion (Higuchi model) [90]. From the plot of log(*Mt*/*M*) versus log(*t*), kinetic parameters, *n,* and *k* were calculated.

*M*_*t*_/*M* = *kt*^*n*^ (1)

Log (*M*_*t*_/*M*) = log *k* + *n* log (*t*) (2)

## Figures and Tables

**Table 1:** Stability values of SDs in the ratio of 1/10 (*w*/*w*) FB:P407.

Measurement	Observed Absorbance Values		
	Deep Freezer (−20 ± 5 °C)	Refrigerator (5 ± 3 °C)	Room Temperature (25 ± 2 °C)
Initially	0.483	0.483	0.483
15th day	0.474	0.476	0.482
30th day	0.479	0.478	0.474
60th day	0.467	0.467	0.469
Mean	0.475	0.476	0.477
SD	0.007	0.007	0.007
CV%	1.45	1.4	1.4

**Table 2:** Gelation temperatures of P407/P188 mixtures containing FB-SDs equivalent to 0.03% FB.

P407/P188%	Gelation Temperature (°C)
15/18	40.4 ± 0.07
15/20	39.1 ± 0.07
15/26	34.4 ± 0.07
15/26.5 (F1)	33.9 ± 0.71
15/27	33 ± 0.07
16/27	34.8 ± 0.14
24/5	33.2 ± 0.15
23/5	36 ± 0.8

**Table 3:** Compositions, gelation time, and gelation temperatures of thermosensitive and bioadhesive ophthalmic formulations containing FB-SDs equivalent to 0.03% FB.

F	P407/P188 %15/%26.5	CP (%)	CMC (%)	Gelation Time (s)	Gelation Temperature (°C)	
					Method 1	Method 2
F1	+	−	−	298	33.9	34.0
F2	+	0.2	−	120	33.3	32.5
F3	+	0.4	−	71	32.6	32.0
F4	+	0.6	−	73	32.4	32.0
F5	+	−	0.2	235	33.2	33.7
F6	+	−	0.4	207	33	33.7
F7	+	−	0.6	150	32.9	33.0

**Table 4:** Mechanical properties of formulations determined using texture analysis at 25 °C and 34 °C.

F	H ± SD(N)	C ± SD(N mm)	A ± SD(N mm)	H ± SD(N)	C ± SD(N mm)	A ± SD(N mm)
		25 °C			34 °C	
F1	0.011 ± 0.00	0.043 ± 0.00	0.039 ± 0.00	0.965 ± 0.48	3.906 ± 0.04	3.642 ± 0.13
F2	0.015 ± 0.05	0.050 ± 0.00	0.044 ± 0.00	1.253 ± 0.18	4.781 ± 0.20	3.920 ± 0.26
F3	0.027 ± 0.11	0.066 ± 0.00	0.105 ± 0.00	1.458 ± 0.49	5.770 ± 0.09	4.679 ± 0.13
F4	0.034 ± 0.30	0.084 ± 0.01	0.145 ± 0.00	1.710 ± 0.98	6.628 ± 0.78	5.031 ± 0.43
F5	0.010 ± 0.05	0.035 ± 0.00	0.032 ± 0.00	1.048 ± 0.00	3.768 ± 0.26	3.494 ± 0.54
F6	0.011 ± 0.02	0.036 ± 0.00	0.029 ± 0.00	1.068 ± 0.50	4.324 ± 0.16	4.133 ± 0.27
F7	0.010 ± 0.03	0.033 ± 0.01	0.029 ± 0.00	1.283 ± 0.50	4.990 ± 0.08	4.949 ± 0.25

F: formulation, H: hardness, C: compressibility, A: adhesiveness.

**Table 5:** Release kinetic parameters of FB from thermosensitive and bioadhesive ophthalmic formulations.

Formulation	Zero-Order		First-Order		Higuchi		Hixson-Crowell	
	r^2^	∑(Resid)^2^/n−2	r^2^	∑(Resid)^2^/n−2	r^2^	∑(Resid)^2^/n−2	r^2^	∑(Resid)^2^/n−2
F1	0.9763	120.4020	0.9896	107.7413	0.9813	83.0534	0.9907	334.9105
F2	0.9785	154.6521	0.9979	15.0035	0.9936	21.9354	0.9962	74.4396
F3	0.9805	95.1133	0.9955	34.5983	0.9893	39.0485	0.9950	159.5296
F4	0.9848	45.9916	0.9780	43.8904	0.9665	66.5108	0.9821	154.6797
F5	0.9772	311.5803	0.9967	24.6281	0.9931	25.8022	0.9964	55.7461
F6	0.9731	131.8692	0.9937	46.0530	0.9856	55.7539	0.9911	184.4683
F7	0.9891	28.7591	0.9932	20.2110	0.9870	33.2258	0.9941	101.2407

**Table 6:** n exponent assessments of release data of FB from thermosensitive and bioadhesive ophthalmic formulations.

Formulation	*n*	*k*	*R^2^*
F1	1.0148	1.009	0.9836
F2	1.0044	0.9829	0.9882
F3	1.0281	0.9644	0.9899
F4	1.0506	0.7744	0.986
F5	0.9323	1.072	0.9911
F6	1.0351	0.9713	0.9873
F7	1.0404	0.8657	0.9957

## Data Availability

All of the data are contained in this manuscript.
